# The Influence of Contralesional Brain Age Gradients on Chronic Stroke Aphasia Severity and Treatment Response

**DOI:** 10.1523/JNEUROSCI.2058-25.2026

**Published:** 2026-10-21

**Authors:** Nicholas Riccardi, Ansley Martin, Ida Rangus, Natalie Busby, Chris Rorden, Julius Fridriksson, Leonardo Bonilha

**Affiliations:** ^1^Department of Communication Sciences and Disorders, University of South Carolina, Columbia, South Carolina 29208; ^2^Center for Stroke Research Berlin, Charité – Universitätsmedizin Berlin, Berlin 10117, Germany; ^3^Department of Psychology, University of South Carolina, Columbia, South Carolina 29208; ^4^Department of Neurology, School of Medicine Columbia, Columbia, South Carolina 29208

**Keywords:** aphasia, brain age, language, MRI, stroke, treatment response

## Abstract

The adult brain follows normative aging trajectories, yet stroke abruptly disrupts this process. Beyond the focal lesion, undamaged regions can deviate from expected aging due to diaschisis or pre-existing vulnerabilities, reflecting tissue health that may constrain recovery. Chronic post-stroke aphasia, a persistent language disorder, shows wide individual variability in severity and treatment response, and these differences are not fully captured by lesion characteristics or demographic factors. Further, prior imaging biomarkers have been difficult to scale because they rely on specialized acquisitions or nonstandardized analytical pipelines. Here, we used a standardized, lifespan-informed brain age model (volBrain) trained on large public datasets to quantify regional deviations from normative aging using only routine structural MRI (T1-weighted). In 188 individuals with chronic stroke (67 female, 121 male), regional aging deviations in the contralesional hemisphere explained aphasia severity beyond lesion volume, lesion location, and basic demographics. In a treated subgroup, baseline brain aging patterns also predicted language gains that persisted 6 months after therapy, indicating prognostic value. By characterizing how individual brains diverge from expected aging, this approach captures variation in tissue integrity that is relevant for both impairment and recovery. This provides a scalable framework that can be integrated across sites and studies, linking normative aging models with post-stroke prognostics and informing precision neurorehabilitation.

## Significance Statement

Stroke can change the brain in ways that extend beyond the visible lesion. This study shows that parts of the brain that appear structurally intact can show signs of accelerated or altered aging and that these changes relate to how severe a person's language impairment is after stroke and how much they benefit from therapy. Using a standardized “brain age” model that works on routine MRI scans, we identified patterns of tissue integrity that explained who had better or worse outcomes, even when accounting for lesion size and location. This approach is scalable and can be used across research and clinical settings. These findings highlight the role of brain aging in recovery and can support more personalized rehabilitation planning.

## Introduction

Chronic aphasia following left hemisphere stroke is a persistent language disorder that substantially impacts quality of life (Hilari et al., 2012). Speech-language therapy is the standard treatment, but individuals show wide variability in response. Group-level gains are well established, yet predicting individual outcomes remains difficult (Doogan et al., 2018).

Lesion-symptom mapping (LSM) has been central for linking lesion location to language deficits. However, lesion characteristics do not fully account for severity or recovery, motivating a paradigm shift toward evaluating the status of the intact, remaining brain (Kiran et al., 2019).

Studies have used functional MRI and diffusion tensor imaging (DTI; Forkel et al., 2014; Stefaniak et al., 2022), providing important insights, but these methods do not fully capture individual variability. People with similar lesion anatomy can differ markedly in the condition of remaining brain tissue due to vascular burden, small vessel disease, or other pre-existing variabilities (Busby et al., 2025; Hannan et al., 2025).

Brain age modeling addresses this gap: models trained on large normative datasets estimate how old a brain appears relative to chronological age (Cole et al., 2017). The difference between estimated and chronological age (brain age gap; BAG) serves as a proxy for tissue health (Franke and Gaser, 2019) and is associated with cardiovascular risk, reduced cognitive performance, and mortality (Cole et al., 2018). Its application to aphasia, however, remains limited (Fig. 1), despite offering a scalable way to characterize tissue health from routine structural MRI.

**Figure 1.**
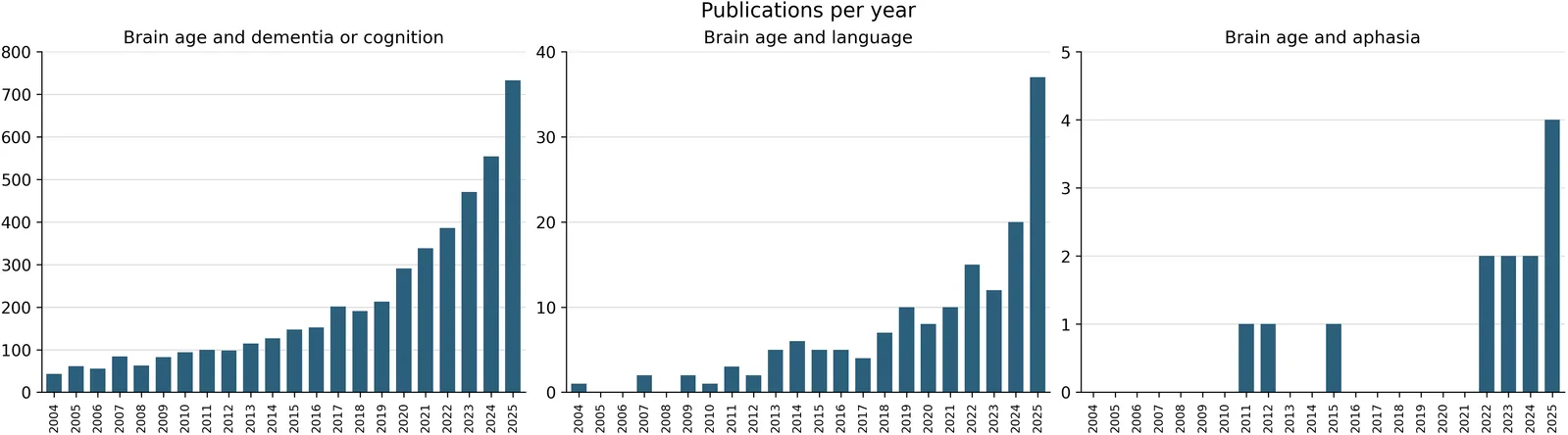
Publications per year (2004–2025) matching three PubMed queries for brain age literature. Left, (“brain age” OR “brain aging”) AND (dementia OR cognition); 4,597 articles. Middle, (“brain age” OR “brain aging”) AND “language”; 155 articles. The quoted “language” bypasses PubMed's automatic medical subject headings (MeSH) expansion (which otherwise pulls in “programming languages”). Right, (“brain age” OR “brain aging”) AND aphasia; 13 articles. Brain age has thousands of publications related to dementia and cognition, with far fewer addressing language and aphasia specifically.

In stroke, older brain age is associated with poorer sensorimotor outcomes and partially mediates the effect of lesion damage on outcome (Liew et al., 2023). Brain age tracks cardiovascular risk and 3-month functional outcome in ischemic stroke (Bretzner et al., 2023). In post-stroke aphasia, brain age has been linked to chronic aphasia severity (Busby et al., 2023, 2024) and to long-term recovery from acute stroke (Kristinsson et al., 2022a). These studies establish that whole-brain age carries signal beyond chronological age in stroke but have largely relied on global (first-generation) estimates and have not examined the chronic phase longitudinally. Whether the regional topography of brain age in spared tissue carries additional information about aphasia severity, and predicts longitudinal chronic-phase outcomes, remains untested.

Brain age is distinct from traditional measures such as gray matter volume. Next-generation brain age models use deep learning to detect multivariate patterns across volume, shape, and intensity and provide region-specific rather than single “global” estimates. This is critical because age-related vulnerability is unevenly distributed, disproportionately affecting association cortices (Raz et al., 2005).

Here, we tested this in 188 individuals with left hemisphere stroke, applying a pretrained regional brain age model (Nguyen et al., 2024) to the intact right hemisphere. We focused on the right hemisphere given evidence that contralateral structure relates to aphasia outcomes (Xing et al., 2016) and to avoid lesion-based influences on regional brain age quantification. We used exploratory factor analysis (EFA) to identify spatial gradients of lesion damage and brain aging. Both processes follow nonrandom anatomical structure: stroke lesions track arterial branches, and regional aging aligns with cortical hierarchies (Riccardi et al., 2025b). EFA can therefore uncover this spatial structure while addressing collinearity (Zhao et al., 2020). Factor scores then index how strongly each individual expresses each pattern.

We had three hypotheses. First, regional brain aging would show interpretable spatial structure (Riccardi et al., 2025b). Second, these aging gradients would be associated with baseline aphasia severity (assessed at chronic-phase study enrollment) beyond lesion features and demographics. Third, aging gradients would provide added predictive value for individual treatment response. In a subset of individuals (*N* = 34) who completed a clinical trial (Fridriksson et al., 2018) of speech-language therapy with adjunctive anodal transcranial direct-current stimulation (A-tDCS), we tested whether pretreatment brain aging explained naming gains 6 months after treatment. To minimize predictor-selection bias, we first modeled baseline naming in an independent group (*N* = 131) that did not receive A-tDCS and retained only significant predictors for the treatment response analysis.

## Materials and Methods

### Participants

This is a retrospective study of data gathered by the Center for the Study of Aphasia Recovery (C-STAR) at the University of South Carolina. It included individuals with a left hemisphere ischemic or hemorrhagic stroke at least 6 months prior to enrollment, and it excluded individuals with previous strokes or those with a history of other neurological conditions (e.g., dementia, Parkinson's disease). In total, 188 participants (67 female) had available neuroimaging, behavioral, and demographic data, collected upon study enrollment in the chronic phase (henceforth “baseline”; Table 1).

**Table 1.** Participant characteristics (*N* = 188)

	Mean or %	SD or N
Demographic Variables		
Sex, female	35.6%	67
Chronological age, years	60.95	10.95
Education: bachelor's or higher^a^	55.9%	105
Stroke variables		
Age at stroke, years	57.96	11.76
Time post-onset, months^b^	49.9	52.5
Lesion volume, cm^3^	121.72	93.15
Aphasia		
WAB-R AQ	60.67	27.09
PNT baseline	81.8	59.26

WAB-R AQ, Western Aphasia Battery-Revised Aphasia Quotient; PNT, Philadelphia Naming Test.

a Education was collected categorically. *N* = 105 participants (55.9%) held a bachelor's degree or higher.

b Time post-onset (*N* = 188): mean ± SD = 49.9 ± 52.5 months; median 26.1 months (IQR 14.8–66.4; range 6.0–262.8 months). All 188 participants were ≥6 months post-onset at enrollment (chronic phase).

A subset of 34 of these individuals participated in the treatment arm of a clinical trial (Fridriksson et al., 2018) that tested adjunctive A-tDCS during 3 weeks of speech therapy. These participants had follow-up language testing 6 months after conclusion of treatment, which we used to examine individual differences in treatment-related gains. All study procedures were approved by the Institutional Review Boards of the University of South Carolina and the Medical University of South Carolina. Written informed consent was obtained from each participant, with a caregiver and speech-language pathologist present to confirm comprehension and voluntary participation.

### Behavioral data

Language was assessed upon enrollment using the Western Aphasia Battery-Revised Aphasia Quotient (WAB-R AQ; Kertesz, 2007) and the Philadelphia Naming Test (PNT; Roach et al., 1996). These were administered and scored by certified speech-language pathologists. AQ scores range from 0 to 100, with higher scores indicating better language abilities. Scores ≤93.8 are indicative of aphasia. To capture the full range of post-stroke language function, all participants were included regardless of whether they met that threshold (*N* = 18 had an AQ > 93.8).

The PNT was administered twice upon enrollment, on separate days within the same week, and was available for 165 participants. The two scores were averaged to account for day-to-day variability in performance and used as the “baseline PNT.” A subset of 34 participants who received adjunctive A-tDCS during 3 weeks of speech therapy again took the PNT twice, 6 months after concluding treatment, with the two scores again averaged. This served as a longitudinal measure of change in naming performance. For these participants, the difference between baseline PNT and 6-month follow-up PNT is henceforth referred to as “treatment change.”

### Adjunctive A-tDCS treatment protocol

Brain stimulation used a constant current stimulator (Phoresor II PM850; Iomed) that administered 1 mA of A-tDCS stimulation induced between two 5 × 5 cm saline-soaked sponges (electrodes). The anode electrode was placed on the left scalp over a targeted cortical region and the cathode electrode was placed on the contralateral supraorbital frontal scalp region (above the right eyebrow). All participants completed two MRI sessions at baseline, which included a picture naming fMRI protocol. The anodal electrode was placed over the temporal lobe region with the highest naming-related activation on the fMRI [for full details, see Fridriksson (2010)]. Each individual's fMRI data were coregistered with their T1w images, and a magnetic position tracker (Ascension Technology flock-of-birds) in combination with MRIreg (http://people.cas.sc.edu/rorden/mricro/mrireg/index.html) was used to coregister each individual's scalp coordinates with their T1w image. The desired cortical region was then located and demarcated on a latex cap worn by the patient. This cap was carefully fitted on the patient's head prior to the start of each tDCS administration to accurately position the anode electrode in the same area from one day to the next. After positioning, the cap was removed, and electrodes were held in place with self-adhesive bandages. A-tDCS stimulation was started at the beginning of the behavioral treatment sessions and remained active during the first 20 min of the 45 min treatment session.

The treatment was performed through a computerized task (Fridriksson et al., 2009, 2011) that involved matching pictures depicting common objects with words that were heard (via headphones) and seen (the face of the speaker below the nose was shown on the computer screen). Patients were instructed to press a green response button if the picture and spoken word matched and a red response button if they did not. Incorrect matches included a semantic foil, a phonological foil, or an unrelated word. Half of the pairs were a correct match. Immediate feedback was provided following each response, and task accuracy was displayed on the computer screen at the end of each session to allow patients to monitor their progress. A total of 160 low-, medium-, and high-frequency words not included on the PNT were used in the task. For full details, see Fridriksson et al. (2018).

### Neuroimaging data

#### Acquisition and preprocessing

Brain scans were acquired in the same week as baseline language testing and, for the 34 A-tDCS participants, prior to the first stimulation session, using Siemens 3 T MRI scanners located at the University of South Carolina, Columbia, SC, or at the Medical University of South Carolina, Charleston, SC. Of the 188 enrollment scans, 61 were acquired using the original 12-channel head coil (all others used the 20-channel coil following an upgrade). volBrain's Brain Structure Ages pipeline is trained and validated on heterogeneous scanner configurations, and all 188 scans passed volBrain's automated quality control and visual inspection. Only T1- and T2-weighted images were used for this study.

T1w imaging used an MP-RAGE sequence with 1 mm isotropic voxels, a 256 × 256 matrix size, a 9° flip angle, and a 192-slice sequence with repetition time (TR) of 2,250 ms, inversion time (TI) of 925 ms, and echo time (TE) of 4.11 ms. A 3D T2 SPACE sequence used a resolution of 1 mm^3^ with a field of view of 256 × 256 mm, 160 sagittal slices, variable degree flip angle, TR of 3,200 ms, TE of 212 ms, and ×2 GRAPPA acceleration (80 reference lines).

#### Lesion tracing and segmentation

Stroke lesions were demarcated by a neurologist (L.B.) in MRIcron on individual T2-weighted images (in native space), blinded to participant performance and demographics. The lesion maps, in T2 space, were coregistered to the participant's T1w with open-source MATLAB scripts (https://github.com/neurolabusc/nii_preprocess; Rorden et al., 2012) and SPM12 (Functional Imaging Laboratory, Wellcome Trust Center for Neuroimaging Institute of Neurology, University College London; http://www.fil.ion.ucl.ac.uk/spm/software/spm12/). Lesion maps in native T1 space were normalized into the Montreal Neurological Institute (MNI) 152 standard brain template (Fonov et al., 2009). Normalization of lesion maps was conducted in steps of the following: (1) smoothing lesion maps by removing uneven edges with a 3 mm full-width at half-maximum Gaussian kernel, (2) binarizing the smoothed lesion maps with a threshold of 0, and (3) enantiomorphic transformation of the participant's T1-weighted image onto stereotaxic space (Nachev et al., 2008). In Step 3, using the *nii_preprocess* pipeline and SPM12's unified segmentation-normalization, a chimeric T1-weighted image was created with a voxel size of 1 mm^3^, where the stroke lesion was replaced by the mirrored equivalent of the intact right hemisphere to create “healed” brains to avoid tissue deformation (Rorden et al., 2012).

After normalization, each participant's binarized lesion mask was intersected with volBrain's atlas (66 left hemisphere parcels). For every parcel, the number of lesioned voxels and the total number of voxels within a parcel were counted, and a lesion proportion score was computed. Scores range from 0 (no damage) to 1 (complete damage). This yielded a 66-element lesion proportion vector for each participant (Fig. 2).

**Figure 2.**
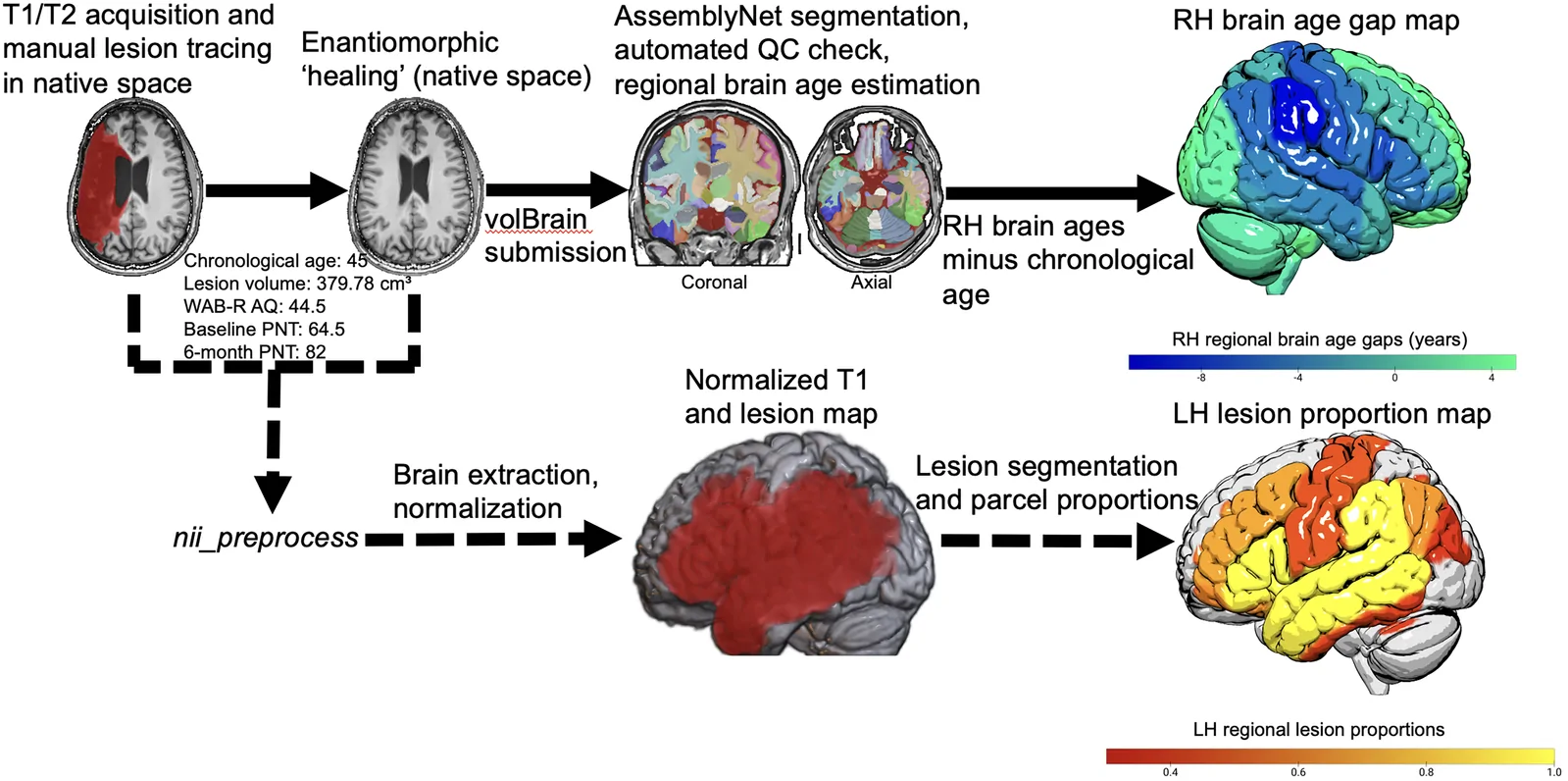
End-to-end image-processing workflow for extracting left hemisphere (LH) lesion metrics and right hemisphere (RH) brain age gaps in an example participant. It requires only T1 and T2 images, and all steps following manual lesion tracing can be automated with publicly available tools. Solid arrows trace the brain age pipeline, resulting in a heterogeneously distributed RH map of parcel-wise brain age gaps. Dashed arrows trace the lesion pipeline, resulting in a LH parcel map colored by lesion proportion. The processing branches converge on two complementary outputs, LH lesion proportions and RH brain ages, that serve as imaging features in subsequent statistical models.

#### Brain age estimation

Parcel-wise brain age estimates for the right hemisphere were calculated using volBrain's Brain Structure Ages (Nguyen et al., 2023, 2024), a pretrained, automated, publicly available pipeline (https://volbrain.net/; Fig. 2). The tool expects raw T1 images in native space. It was trained and validated on brains without stroke lesions.

Importantly, regional brain age was calculated based on the enantiomorphically “healed,” de-identified (defaced) T1 scans described above. That is, each participant's lesions were replaced with the mirrored tissue from the contralesional hemisphere, but the image otherwise remained unprocessed and in native space. This chimeric repair enables the pipeline to run successfully across the entire brain, but the replaced tissue renders age estimates in the altered left hemisphere not valid for interpretation. Therefore, all brain age analyses were restricted to right hemisphere parcels. Importantly, volBrain's segmentation and parcel-wise brain age estimation (described below) do not require substantial information to be mixed across hemispheres, so the chimeric left hemisphere voxels do not contaminate parcel-wise brain age estimates for the right hemisphere.

The only study-specific preprocessing step was the enantiomorphic lesion repair described above. All subsequent processing was performed entirely by volBrain's automated and publicly available pipeline with no modifications. As described in volBrain's documentation (Manjón and Coupé, 2016; Coupé et al., 2020; Manjón et al., 2022; Nguyen et al., 2024), Brain Structure Ages uses a multistep deep learning process to estimate brain ages. First, the brain is parcellated into 132 regions with AssemblyNet (Coupé et al., 2020), an ensemble of 3D U-Net models trained to replicate manual segmentations of brain regions made by expert neuroanatomists and radiologists according to the BrainCOLOR protocol. It integrates multiple overlapping subvolumes, improving accuracy of segmentation by leveraging strengths of different U-Net models. A different U-Net ensemble was trained to predict chronological age at the voxel level from raw T1 images. They used convolutional and deconvolutional layers to progressively learn features in the MR image that are most predictive of chronological age without requiring predefined morphometric properties like GMV or cortical thickness. This results in a heterogeneous map of parcel-wise age estimates for each individual.

Brain Structure Ages was trained and validated on thousands of MRIs from multiple datasets (e.g., UK Biobank), with individuals aged 5–85. During model training, each T1 image (registered to MNI space as part of volBrain's pipeline) was first downsampled by a factor of two, resulting in a 91 × 109 × 91 voxel image. Then, overlapping subvolumes (size 32 × 48 × 32 voxels) were extracted across the downsampled image. A total of 125 subvolumes were spaced along the three spatial dimensions. A separate U-Net model was trained by each subvolume to predict chronological age, resulting in 125 independent U-Net models which each contributed to a reconstructed 3D age map. For regions where voxels appeared in overlapping subvolumes, predictions were averaged to reduce variability. The 3D age map was then upscaled with trilinear interpolation to match the original MRI resolution and was used to calculate parcel-level brain age estimates.

During training, voxels within the intracranial cavity (ICC) were given the participant's chronological age as “ground truth,” and voxels outside the ICC were set to zero. Models were refined with stochastic gradient descent with the mean absolute error as the loss function. Batches of eight images were used for training, with a stopping criterion of 20 epochs if no improvement was seen in validation loss. The first U-Net was trained from scratch, while subsequent models were initialized with transfer learning from adjacent U-Nets. Data was split into 80% training and 20% validation sets. When a new U-Net was trained, the split was redefined to maximally use available data. To avoid overfitting, random spatial shifts and mix-up augmentation were used, which improves generalization by combining patches from different participants. To validate the age prediction in an independent test set, regional brain ages of the training participants were computed. These regional brain ages were then used to train a support vector regression-based model with 10-fold cross-validation, resulting in 10 individual models. Each testing sample was evaluated with all 10 models, and age estimates were averaged to facilitate generalization. Two independent datasets were used to measure model accuracy, with the pipeline achieving accuracy in older adults that was comparable to or better than other well-validated brain age models (MAE = 3.83 years). Note that a “perfect” brain age estimation model is not desirable, as it is the deviation from normative aging which provides meaningful information about brain structure.

### Statistical analyses

#### Characterizing stroke lesion patterns and brain age gradients

Motivated by prior work using dimension reduction on lesion data (Fridriksson et al., 2016; Zhao et al., 2020; Bonkhoff et al., 2021), we used EFA to reduce the dimensionality of our neuroimaging data for theoretical and practical reasons. First, with 188 participants but 132 regional values per participant, the dataset is wide. Machine learning approaches in such settings remain sensitive to “small” *N* (in the context of ML), heterogeneous lesion coverage, and spatial multicollinearity. These challenges are especially pronounced in stroke research (Mah et al., 2014; Sperber and Karnath, 2018; Ivanova et al., 2021; Teghipco et al., 2024), where damage is often sparse and structurally correlated. EFA offers an alternative by reducing correlated parcels into a smaller number of factors, revealing dominant spatial patterns, improving interpretability, and reducing model instability.

Second, vascular anatomy constrains infarct location (Olsen et al., 1985; Phan et al., 2007; Liu et al., 2023). Lesions are not randomly distributed across the brain. Rather, they follow arterial branches, meaning that neighboring parcels are often damaged together, resulting in strong spatial collinearity. While this presents a challenge for univariate LSM (Inoue et al., 2014; Sperber and Karnath, 2016, 2017, 2018), it is well suited to EFA, which leverages collinearity to identify patterns of codamage that should reflect underlying vascular structure of the brain. The resulting lesion patterns are a data-driven summary of commonly codamaged regions. Similarly, for brain age, parcel-wise variability is not independent but is instead organized along gradients that govern system-level development and atrophy throughout the lifespan (Burt et al., 2018; Huntenburg et al., 2018; Sydnor et al., 2021; Riccardi et al., 2025a,b). In both contexts, EFA provides a principled way to uncover spatially structured and biologically grounded dimensions of variability.

Third, each factor is bidirectional. A greater positive participant-level factor score means that an individual has a topological motif that aligns with an identified factor, with damage or accelerated aging in high-loading factor regions and no damage or decelerated aging in low-loading factor regions. A greater negative participant-level factor score would indicate an “opposite” pattern, with sparing or decelerated aging in high-loading factor regions. This signed, continuous measure supports two-sided testing of behavioral associations with either damage or preservation along a factor pattern.

Here, two independent EFAs were conducted, one on LH lesion proportions and one on RH brain age gaps, and stability checks to ensure robustness and replicability of the resulting factors were applied.

*Lesion EFA*. After processing, each participant's LH was represented by a 66-element lesion proportion vector. To avoid instability due to regions that are rarely lesioned in our cohort, we excluded the ventricles and parcels that had ≤5% damage in ≤10% of participants, leaving 48 parcels for analysis. Lesion proportions for each region were logit-transformed after clipping values to the range (0.001–0.999) to spread values that were previously compressed near 0 or 1, making them more suitable for EFA. Total lesion volume was log-transformed to normalize its distribution and then *z*-scored. We regressed this transformed lesion volume from each parcel to ensure that extracted factors reflected spatial damage patterns and not simply lesion volume effects. Residual values were then *z*-scored across participants to standardize each parcel's scale prior to EFA.

In SPSS v29.0.1, we performed principal axis factoring on the resulting correlation matrix, with varimax rotation and Kaiser normalization. Sampling adequacy was good (KMO = 0.86), and Bartlett's test confirmed that the matrix contained sufficient common variance (*χ*^2^ = 12,122.6, *p* < 0.001). Factors were extracted based on an eigenvalue >1, resulting in eight factors that explained 82.8% of variance.

To evaluate robustness of the eight factors, we conducted 1,000 random split-half resampling iterations of the EFA using Python's FactorAnalyzer. For each iteration, we randomly divided the 188 participants into two equal groups (*N* = 94 per group), repeated the full factor extraction within each half, and then compared the two solutions. Because the order of factors in EFA is not meaningful beyond the amount of variance explained, and can vary across solutions, we applied Hungarian assignment to align columns of the two factor loading matrices based on their loading patterns. This finds the one-to-one matching that maximizes the overall absolute correlation between factor loadings. This ensures that, for example, factor 1 in split A is aligned with its closest counterpart in split B. After the factors were matched, we quantified their similarity using Tucker's congruence coefficient (*ϕ*; Korth and Tucker, 1975; Lorenzo-Seva and ten Berge, 2006; Lovik et al., 2020). This compared the entire pattern of a factor's loadings across the two splits. It ranges from −1 to 1 and is sensitive to even small differences between factor loadings. It is more conservative than other approaches that could be used to measure factor congruence. While *ϕ* > 0.8 is a typical threshold to determine if two factors are congruent, we elected to use the more stringent threshold of *ϕ* ≥ 0.85 as recommended by Lorenzo-Seva and ten Berge (Lorenzo-Seva and ten Berge, 2006). Thus, a factor was deemed stable if, across the 1,000 split-half replications, it achieved *ϕ* ≥ 0.85 in at least 60% of splits. Six of the eight factors met these criteria and were retained. Participant-level factor scores, representing how closely an individual's lesion pattern matches each of these six factors, were calculated with SPSS's regression method and retained for further analysis.

*Brain age EFA*. Each participant's RH was represented by a 66-element vector of brain age gaps (estimated parcel age minus chronological age). We excluded the two ventricle age estimates from analysis, resulting in a 64-element vector. No additional transformations were applied. EFA identical to the above was then conducted. Sampling adequacy was good (KMO = 0.97), and Bartlett's test confirmed that the matrix contained sufficient common variance (*χ*^2^ = 50,171.3, *p* < 0.001). Four factors were retained, and they explained 96.1% of the variance. The same split-half resampling as described above confirmed that all four RH brain age factors were stable.

#### Do brain age gradients explain additional variance in aphasia severity?

Prior to analyses, we confirmed that participant-level factor scores were orthogonal by computing pairwise Pearson’s correlations separately for the lesion factors and brain age factors. Further, all subsequent models were inspected for collinearity (e.g., tolerance and VIF) and were within acceptable ranges (all tolerances >0.3).

In all 188 individuals, we used three-block hierarchical (enter) regression to explain variance in aphasia severity (WAB-R AQ). Block 1 consisted of standard clinical covariates: chronological age, biological sex, and total lesion volume. Block 2 consisted of participant scores for the lesion factors. Block 3 was the participant scores for the brain age factors. These were added last to test whether intact hemisphere tissue health provides incremental predictive value beyond global variables and lesion location. Predictor position in the final block isolates their unique contribution and avoids conflating them with lesion-related variance. All models were run in SPSS v29 and evaluated with 1,000 bias-corrected and accelerated (BCa) bootstrap replications. In identical fashion, we also conducted exploratory modeling of the four main WAB-R subtests (naming, comprehension, repetition, and spontaneous speech), though WAB-R AQ was our primary focus.

#### Do brain age gradients explain additional variance in treatment response?

To isolate treatment-specific effects without inflating Type I error, we adopted a two-step, split-sample procedure. First, we analyzed baseline PNT scores in participants who did not receive A-tDCS (Group 1, *N* = 131). A three-block hierarchical regression identical to the above identified the predictors that were significantly related to baseline naming. Only the significant variables were retained for the second step, where we used those preselected predictors to model naming change in the independent A-tDCS cohort (6-month PNT minus baseline PNT; Group 2, *N* = 34), with coefficient estimates obtained via BCa bootstrap.

Note that the lesion and brain age factors were obtained from an unsupervised factor analysis run on the entire MRI dataset, and no behavioral scores were used in that step. Factor scores are simple linear combinations of each participant's parcels, so using these factor weights does not pass any outcome (behavioral) information from the tDCS group back into the predictor selection that was used in Group 1. This keeps the two groups independent while still letting us reduce the number of predictors for the smaller tDCS sample.

## Results

### Identifying stroke lesion patterns and brain age gradients

#### Lesion EFA

EFA with split-half resampling revealed six stable patterns that explained variance in the lesion data and resembled discrete vascular territories (Fig. 3*A*,*B*): (1) inferior frontal, 13.4%; (2) occipital, 12.8%; (3) temporal, 11.3%; (4) parietal, 10.4%; (5) dorsomedial frontal, 8.1%; and (6) subcortical, 8%. These are henceforth referred to as Lesion Factors 1 through 6 (LF1 to LF6). Factor names refer to the dominant anatomical loading rather than arterial territory because individual factors carry partial loadings on regions outside any single artery's boundary. The same naming convention is applied to the brain age factors. Pairwise Pearson's correlations between the participant-level scores for all factors revealed that they are orthogonal (*r* range: −0.04 to 0.04; uncorrected *p*'s > 0.6).

**Figure 3.**
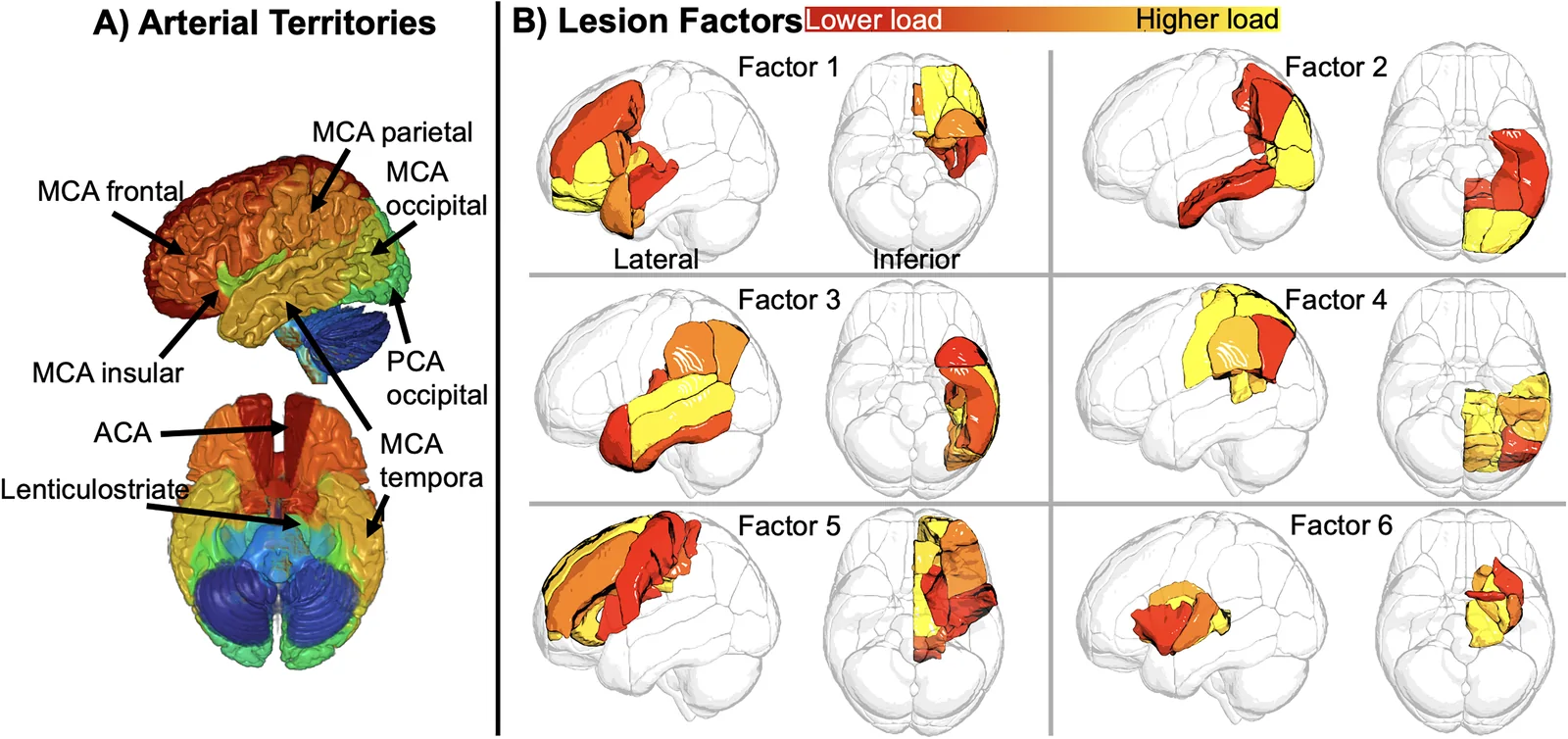
***A***, Arterial territories derived from diffusion-weighted imaging data shared by a separate study of 1,298 patients with acute stroke (MCA, middle cerebral artery; PCA, posterior cerebral artery; ACA, anterior cerebral artery; Liu et al., 2023). Image generated by the present authors. ***B***, Six distinct lesion patterns identified by EFA in our 188 participants with chronic stroke. Yellow indicates higher regional loading in a factor. For example, participants with high LF1 scores would have a stroke lesion which resembles that pattern, with damage to inferior frontal cortices and relative sparing of temporal, parietal, and occipital lobes. Complete regional loading values for each factor can be found in the supplemental material.

#### Brain age EFA

Four stable patterns of brain aging were identified (Fig. 4): (1) medial prefrontal, 35%; (2) fronto-temporo-parietal (language homologs), 30.9%; (3) cerebellar/ventral, 20.7%; and (4) dorsal, 9.2%. These are henceforth referred to as Brain Age Factors 1 through 4 (BAF1 to BAF4). Again, pairwise Pearson's correlations between the participant-level factor scores confirmed orthogonality (*r* range: −0.03 to −0.01; uncorrected *p*'s > 0.7). Example participants can be seen in Figure 5.

**Figure 4.**
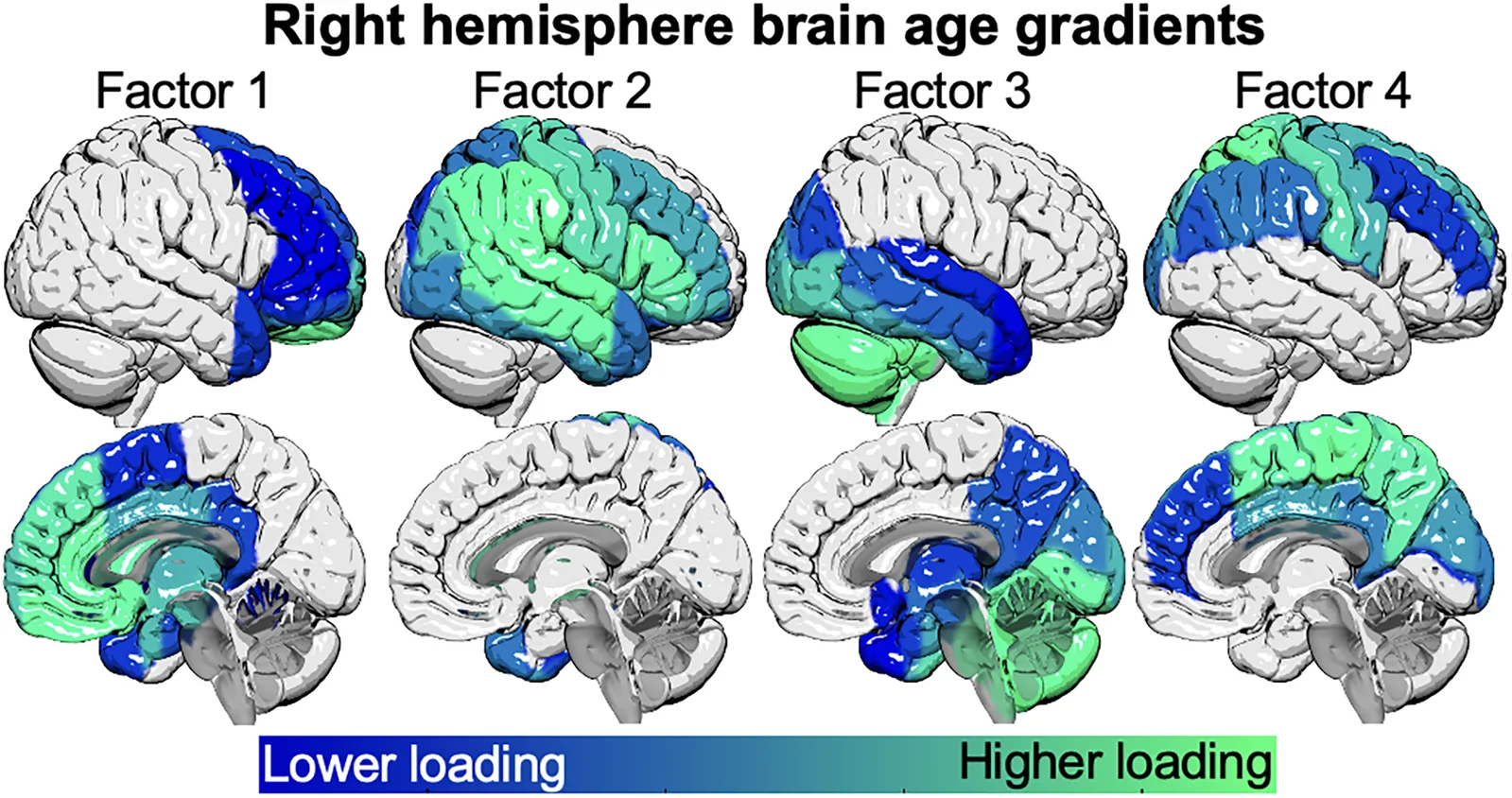
Four right hemisphere brain age patterns that were identified. Green indicates higher regional loading in a factor. For example, participants with high BAF4 scores would have brain aging which resembles that pattern, with relatively accelerated aging in the dorsal stream and relatively decelerated aging elsewhere. Complete regional loading values for each factor can be found in the supplemental material.

**Figure 5.**
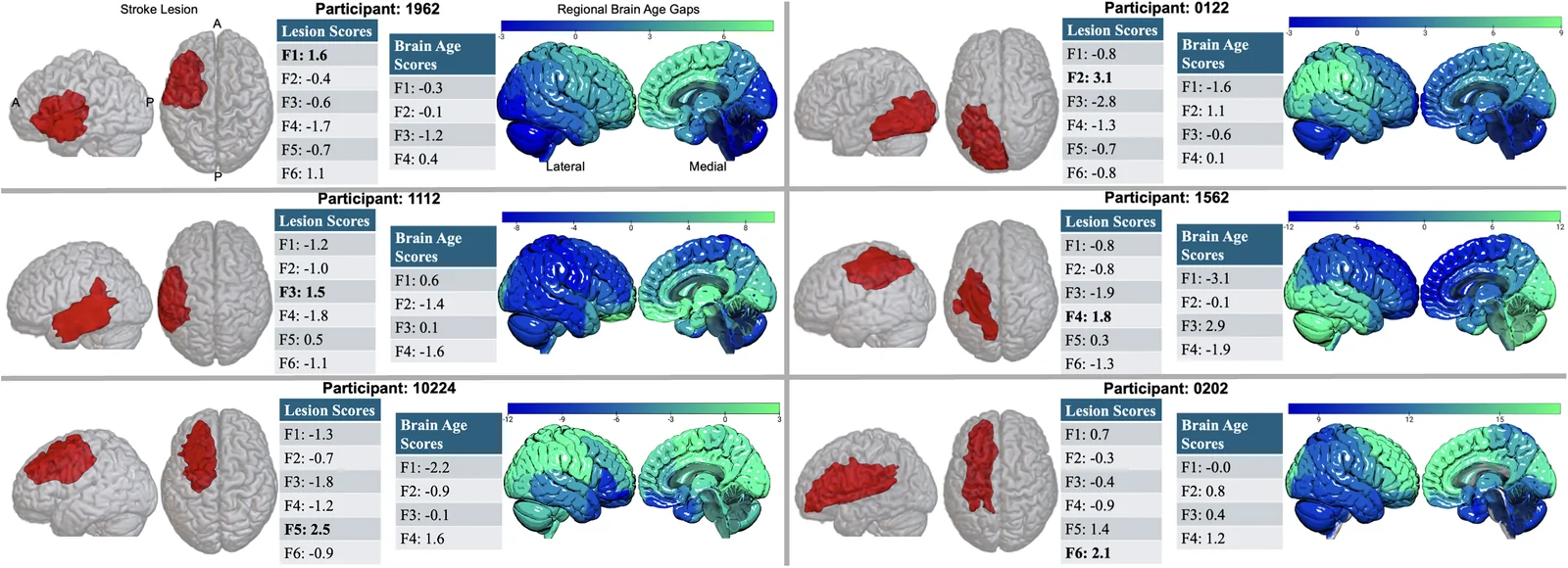
Example participants for factor inspection. Brains on the left of each cell show an individual's stroke lesion in red. Brains on the right show parcel-wise brain age gaps in the contralesional (right) hemisphere, with green areas indicating relatively accelerated brain aging. Tables show each individual's factor scores. Bold values indicate that individual's highest positive lesion factor score. While individual participants can have high or low values for multiple factors, the factor scores are orthogonal at the group level. Note that lesion volume is regressed out from lesion data prior to EFA. This means that participants with large stroke lesions tended to have middling-to-high scores for the factors that their lesions covered, with their primary variance being captured by the lesion volume variable in subsequent analyses.

### Do brain age gradients explain additional variance in aphasia outcomes?

#### WAB-R AQ and subtests (*N* = 188)

“Blocks” here refer to sequential sets of predictors entered into hierarchical regression models. WAB-R AQ: Block 1 explained a significant proportion of variance (adj. *R*^2^ = 0.33, *p* < 0.001). Block 2 (lesion factors) significantly improved model fit (Δ*R*^2^ = 0.05, *p* = 0.037), as did Block 3 (brain age factors; Δ*R*^2^ = 0.07, *p* < 0.001). Significant coefficients were chronological age (*β* = −0.3, *p* < 0.001), lesion volume (*β* = −0.57, *p* < 0.001), LF3 (*β* = −0.1, *p* = 0.05), and right hemisphere BAF1 (*β* = −0.31, *p* < 0.001) and BAF3 (*β* = −0.18, *p* = 0.009). The final model explained 46% of variance in AQ (*R*^2^ = 0.46, adj. *R*^2^ = 0.42, *p* < 0.001). Older participants with larger lesions, lesions in the temporal lobe, and accelerated right hemisphere aging in medial prefrontal cortices or cerebellum/ventral stream tended to have lower AQ scores. Similar results were found with WAB-R subtests (Table 2).

**Table 2.** WAB-R AQ, WAB-R subscores, and PNT models

Panel A. Model fits						
	WAB-R AQ (*N* = 188)	WAB-R Comp.	WAB-R Naming	WAB-R Repetition	WAB-R Spont. Speech	PNT (*N* = 131)
Base model adj. *R*^2^: lesion volume, age, sex	0.33***	0.25***	0.25***	0.27***	0.32***	0.22***
+Lesion factors (Δ*R*^2^)	0.05*	0.06*	0.06*	0.08**	0.04	0.06
+Brain age factors (Δ*R*^2^)	0.07***	0.07***	0.06**	0.06**	0.06***	0.07*
Final adj. *R*^2^	0.42***	0.35***	0.33***	0.38***	0.40***	0.29***
Panel B. Significant coefficients (final model)						
	AQ *β*	Comp. *β*	Naming *β*	Repetition *β*	Spont. Speech *β*	PNT *β*
Chronological Age	−0.30	−0.22	−0.33	−0.29	−0.29	−0.35
Lesion Volume	−0.57	−0.49	−0.50	−0.53	−0.56	−0.42
Lesion factor 2				0.10		
Lesion factor 3	−0.10	−0.15		−0.19		
Lesion factor 5				0.13		
Brain age factor 1	−0.31	−0.27	−0.27	−0.28	−0.30	−0.30
Brain age factor 3	−0.18	−0.17		−0.17	−0.17	

A, Model fits for baseline WAB-R AQ, PNT, and additional WAB-R subtests. Asterisks indicate significance levels: *p* < 0.05*, *p* < 0.01**, *p* < 0.001***. B, Significant predictors for each model, based on BCa bootstrapped confidence intervals (two-tailed).

#### Baseline PNT in the non-tDCS cohort (*N* = 131)

To create a minimally biased predictor set for the upcoming analysis of naming changes in the A-tDCS cohort, we first modeled baseline PNT scores in the group that did not receive adjunctive A-tDCS (Table 2).

Block 1 explained a significant proportion of variance (adj. *R*^2^ = 0.23, *p* < 0.001). Block 2 did not significantly improve model fit (Δ*R*^2^ = 0.06, *p* = 0.15). Block 3 significantly improved model fit (Δ*R*^2^ = 0.07, *p* = 0.01). Significant coefficients were chronological age (*β* = −0.35, *p* = 0.003), lesion volume (*β* = −0.42, *p* < 0.001), and BAF1 (*β* = −0.3, *p* = 0.008). The final model explained 36% of variance (*R*^2^ = 0.36, adj. *R*^2^ = 0.29, *p* < 0.001).

We therefore selected these three variables (chronological age, lesion volume, BAF1) for the A-tDCS naming change model below, hypothesizing that younger chronological age, smaller lesions, and lower right hemisphere BAF1 would relate to larger naming gains.

#### Six-month naming change in the A-tDCS cohort (*N* = 34)

First, a paired-samples *t* test revealed that participants had significantly higher PNT scores at 6-month follow-up (*M* = 68.2, SD = 46.3) than at baseline (*M* = 60.2, SD = 41.5), *t*_(33)_ = −2.7, *p* = 0.012. However, within this group, there was significant individual variability in treatment-related response. For example, 35.3% had a treatment change of ≤0, indicating nonresponse, while 47.1% improved by more than 10 items at follow-up. Here, we attempt to explain this variability. Of note, this represents the subsample of the participants included in the original tDCS study. The results reported here are therefore related to that subsample, but the formal conclusions about that clinical trial are the data presented in the original article (Fridriksson et al., 2018).

The single-block enter regression model including chronological age, lesion volume, and brain age factor 1 explained 23% of variance in naming score change (*R*^2^ = 0.23, adj. *R*^2^ = 0.15, *p* = 0.051; Fig. 6). Because the hypotheses for the individual variables were directional, coefficient significances were evaluated with one-tailed tests (all previous analyses used two-tailed tests). The coefficients for all three independent variables were significant: chronological age (*β* = −0.72, *p* = 0.003), lesion volume (*β* = −0.31, *p* = 0.014), and BAF1 (*β* = −0.47, *p* = 0.03).

**Figure 6.**
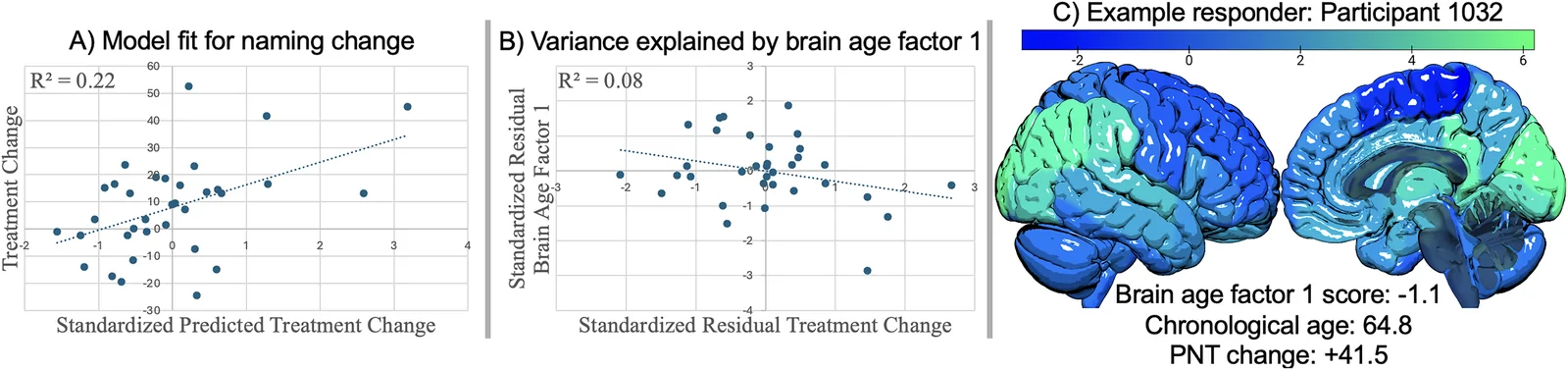
***A***, Observed treatment change (*y*) plotted against predicted values (*x*) from the model including chronological age, lesion volume, and brain age factor 1. ***B***, Partial regression plot showing the relationship between brain age factor 1 scores and treatment change, after controlling for chronological age and lesion volume in both variables. Individuals who more strongly expressed the brain aging pattern of factor 1 (accelerated medial prefrontal aging) did not respond well to treatment, as measured by 6 month follow-up. ***C***, Right hemisphere brain age gap map for an example responder. Brighter green indicates accelerated brain aging. Note the absence of excessive medial prefrontal aging that is characteristic of brain age factor 1.

Considering prior work suggesting that baseline aphasia severity is related to treatment change, we then reran the model twice, adding either WAB-R AQ or baseline PNT as a fourth predictor. Neither baseline variable improved fit (AQ model: *R*^2^ = 0.24, adjusted *R*^2^ = 0.13, *p* = 0.09; PNT model: *R*^2^ = 0.23, adjusted *R*^2^ = 0.12, *p* = 0.10), and BAF1 remained a significant independent coefficient in both cases. Thus, the brain age gradient contributes explanatory power beyond what can be attributed to initial language severity.

To test whether variance in tDCS treatment changes could be explained by gross right hemisphere GMV instead of the specific brain age pattern, a single-block enter regression was used with lesion volume, chronological age, and right hemisphere GMV (residualized on total intracranial volume) as the independent variables. The model was not significant (adj. *R*^2^ = 0.11, *p* = 0.34), suggesting that the brain age pattern is more informative than gross right hemisphere GMV.

A region-matched comparison (a separate EFA on the same 64 right hemisphere regional gray matter volumes, with the resulting factors substituted for the brain age factors in each of the three models) is reported in Supplementary Section S2. The volume EFA did not yield split-half-stable factors, and no volume factor reproduced the BAF1 treatment response effect in the A-tDCS cohort. Exploratory single-block models replacing BAF1 with each of BAF2, BAF3, and BAF4 in turn are reported in Supplementary Materials (Table S1); no other pattern of accelerated brain aging significantly predicted treatment change.

## Discussion

Regional brain age in the intact right hemisphere of stroke survivors explains individual differences in aphasia severity and treatment response beyond demographics and lesion characteristics. This method requires only routine T1-weighted MRI and uses an automated, public pipeline trained on normative aging patterns from thousands of individuals (Nguyen et al., 2024), addressing a long-standing barrier in the field (Boyd et al., 2017): many putative stroke recovery biomarkers depend on specialized acquisitions or pipelines that limit replication across sites.

### Stroke lesion patterns

Brain damage following stroke is nonrandom (Phan et al., 2007). Infarcts are constrained by vasculature, so neighboring regions are often damaged together. Traditional LSM does not explicitly model this structure. We applied an alternative method that embraces this spatial collinearity to identify common lesion motifs across individuals.

This strategy fits a broader effort to summarize stroke heterogeneity in biologically interpretable form. Latent lesion categories have been identified from acute stroke MRIs and captured variance in functional outcomes (Bonkhoff et al., 2021), and related work in chronic aphasia identified lesion components that mapped onto symptom clusters (Zhao et al., 2020). The six lesion factors that we report add to this body of work and provide loadings that can be compared across cohorts.

Our use of EFA reflects a different aim than multivariate lesion-symptom mapping (mLSM; Yourganov et al., 2015; Ivanova et al., 2021). mLSM analyzes all voxels against a specific behavioral target and is optimized for spatial localization given that outcome. EFA instead summarizes lesion anatomy into a set of interpretable factors before any outcome enters the model. The resulting factor scores can therefore be used across outcomes and cohorts without refitting the anatomical decomposition. The trade-off is that the two-step structure (anatomical decomposition, then behavioral validation) may propagate uncertainty into downstream analyses relative to a single-stage mLSM. Here, we used split-half resampling to address factor stability. Additionally, our two-outcome design (baseline and treatment response) favored EFA for practical reasons. Namely, factor scores fit on the full cohort could be reused in the smaller treatment subset (*N* = 34), whereas multivariate analysis faces known stability constraints in smaller samples (Pustina et al., 2018; Sperber et al., 2019; Ivanova et al., 2021).

### Brain age gradients

The identification of spatially structured brain age gradients in the intact right hemisphere is novel. Brain age has been widely used in aging and dementia research (Cole et al., 2019) but is comparatively unexplored in stroke and aphasia. Prior work has typically relied on a single “global” metric that obscures the topographical heterogeneity (Franke and Gaser, 2019). We identified four robust patterns, each capturing distinct topographical variance and significantly associated with language performance. Because these gradients quantify spared tissue integrity rather than lesion damage, they capture interindividual variability that lesion-based methods miss.

### Brain age gradients explain variance in baseline aphasia severity

Before adding brain age, chronological age, lesion volume, and the lesion factors accounted for 38% of the variance in WAB-R AQ, with similar patterns for WAB subtests. LF3, indexing damage centered on the temporal lobe and especially the superior temporal gyrus, was the primary lesion factor, consistent with the established role of temporal regions in language (Hickok and Poeppel, 2007; Bonilha et al., 2017). The benefit of this representation is not predictive gain over voxel-wise LSM (∼40% of variance is typical; Hope et al., 2013; Kristinsson et al., 2021; Riccardi et al., 2024a; Teghipco et al., 2024) but interpretation: mapping language outcomes to a set of vascularly constrained lesion patterns.

Adding brain age gradients explained an additional 7% of variance beyond traditional predictors and lesion factors, exceeding the lesion factor block itself (Δ*R*^2^ = 0.05). In the final model, BAF1's standardized coefficient (*β* = −0.31) was equivalent in magnitude to chronological age (*β* = −0.30), and worse AQ scores were associated with accelerated aging in medial prefrontal cortex (BAF1) and cerebellum/ventral stream (BAF3). Medial prefrontal cortex is a default-mode hub that supports executive aspects of language (Margulies et al., 2016), and portions of the cerebellum support language directly or via cerebellar-cerebral connectivity (Guell et al., 2018). The lesion and brain age findings together describe an interpretable patient profile associated with less severe aphasia: younger individuals with smaller lesions sparing the left superior temporal gyrus and with relatively “younger” medial prefrontal cortex and cerebellum.

This can be interpreted within prior work on the right hemisphere after stroke. fMRI studies have implicated perisylvian regions that are homologs of left hemisphere language cortex (Newport et al., 2017; Wilson and Schneck, 2021; Martin et al., 2024). Structural aging, presented here, may index vulnerability of supporting systems rather than recruitment in service of any specific task. That is, the perisylvian activity from prior work and our structural findings may represent complementary signal. Medial prefrontal cortex aligns with default-mode or domain-general networks that are dissociable from core language cortices (Fedorenko et al., 2024), and BAF1's emphasis on this territory is consistent with degraded domain-general brain health that may contribute to language (or other functions) more broadly.

### Brain age gradients explain variance in individual treatment response

Explaining individual variability in treatment response remains a difficult goal in aphasia, and generalizable prognostic factors are rare (Kristinsson et al., 2022b). To minimize predictor-selection bias, we identified predictors of baseline PNT (chronological age, lesion volume, right hemisphere BAF1) in 131 individuals without A-tDCS and then applied them to model naming change in an independent A-tDCS group at 6 month follow-up (Fridriksson et al., 2018).

This model accounted for 23% of the variance in naming gains. BAF1 remained a significant negative predictor after controlling for chronological age, lesion volume, and baseline aphasia severity. Holding other variables constant, patients whose right hemispheres expressed BAF1 showed little benefit from therapy, and those with the reverse pattern tended to improve. The effect size of BAF1 rivaled chronological age and lesion volume, possibly the most reliable predictors of treatment response (Kristinsson et al., 2022b).

### Limitations and future directions

This study did not aim to build the most comprehensive multimodal model of aphasia outcomes; we focused on the intact hemisphere, using an automated, openly available brain age tool to identify interpretable and reproducible markers of severity and treatment response.

Our analyses used T1-derived morphometrics and do not directly model white matter tracts or lesion-induced disconnection. Disconnection metrics from tractography or connectome LSM (Gleichgerrcht et al., 2017; Riccardi et al., 2024b) could complement this approach: right arcuate fasciculus has been linked to aphasia recovery in longitudinal tractography (Forkel et al., 2014; Osa García et al., 2020), and bilateral white matter connectivity has been characterized across acute and chronic post-stroke aphasia (Schevenels et al., 2022). A natural extension is to test whether disconnection, or a diffusion-based connectome age, adds unique variance beyond the brain age factors used here. Future work should likewise validate rsfMRI brain age as a complementary functional-connectivity approach.

We restricted brain age to the intact right hemisphere because virtually all published brain age models (including volBrain, used here) are trained on lesion-free brains, so left hemisphere estimates would be confounded by destroyed tissue. Masking lesioned parcels avoids tissue replacement but yields heterogeneous parcel coverage across participants that is difficult to model at the group level. Recent work shows that lesion-distorted inputs produce systematically biased brain age estimates and that enantiomorphic repair (used here) corrects this bias (Jeakle et al., 2026).

All imaging and language data were collected in the chronic phase; acute-phase language and imaging were unavailable for this cohort, although acute severity predicts later language outcomes (Pedersen et al., 1995; El Hachioui et al., 2013). Controlling for chronic-phase severity at enrollment and before therapy did not alter the treatment response findings, suggesting that brain age captures variance not redundant with pretreatment severity.

The treatment change measure (6 month minus baseline PNT) spans the 3 week stimulation period and a longer postintervention interval, and BAF1 is a single prestimulation metric from chronic-phase enrollment. Because participants were chronic (≥6 months post-onset, mean 49.9 months), when spontaneous recovery has largely plateaued within ∼10 weeks of onset (Pedersen et al., 1995), the findings are best interpreted as a pretreatment brain age metric explaining individual variability in 6 month outcome following a 3 week intervention, not an index of immediate response.

We did not evaluate the comparative efficacy of A-tDCS; this group served as a test case for whether brain age explains individual variability in treatment response within a rigorously designed trial. Future work should extend this framework to other therapies and stimulation protocols.

### Conclusion

We show that routine structural MRI, analyzed with novel brain age tools, can yield scalable, biologically coherent markers that explain variance in chronic aphasia and predict treatment gains longitudinally. Although validated in one treatment context, these automated biomarkers could also increase power in clinical trials by quantitatively stratifying patients on predicted recovery potential. By sharing the factor loading matrices and scoring coefficients, we invite other groups to test and refine these patterns in diverse cohorts and treatment paradigms, moving the field toward comparable models of post-stroke recovery (https://github.com/NickRiccardi/brain_age_factors).

## Data Availability

Neuroimaging data is publicly available: doi:10.18112/openneuro.ds004884.v1.0.1. All pipelines for reproducing lesion quantification and brain age estimations are publicly available (https://github.com/neurolabusc/nii_preprocess/blob/master/nii_preprocess.m; https://www.volbrain.net/home). Code for generalizing brain age factor scores to new participants is publicly available: https://github.com/NickRiccardi/brain_age_factors.
